# Estimating Inulin Intake and Its Contribution to Total Fibre Intake in UK School‐Aged Children: A Pilot Feasibility Study

**DOI:** 10.1111/nbu.70022

**Published:** 2025-08-13

**Authors:** Gabriela Morillo‐Santander, Christine Ann Edwards, Ada Lizbeth Garcia

**Affiliations:** ^1^ Human Nutrition, School of Medicine, Dentistry and Nursing University of Glasgow Glasgow UK

**Keywords:** children, diet estimates, dietary fibre, fructan, inulin

## Abstract

Inulin is a prebiotic fructan‐type fibre found in vegetables, cereals, and fruits, while isolated inulin is used as a sugar replacement additive. Children in the UK do not meet dietary fibre recommendations, and inulin could contribute to increased fibre intake. However, inulin intake is not routinely assessed. We tested the feasibility of identifying dietary sources of inulin in school‐aged children and estimated the impact of inulin on their fibre intake. In a pilot cross‐sectional study in 154 healthy school‐aged children (median age 7 years old, IQR: 5–12), diet was assessed using one 24‐hour recall. A list of foods reporting inulin content was collated from the literature and food labels. Inulin consumed from homemade and takeaway food was calculated using the mean of three standard recipes. AOAC fibre was estimated using Nutritics software. The UK National Diet and Nutrition Survey (NDNS) was used for comparisons with the estimated fibre‐plus‐inulin intake. Median fibre‐plus‐inulin intake was 16.3 g/day, IQR: 0.9–46.5; AOAC: 985.29 fibre was 14 g/day, IQR: 0.8–45.4 and median inulin intake was 1.3 g/day, IQR: 0.1–7. Fibre‐plus‐inulin estimates were higher than total fibre reported by NDNS in children aged 4–10 years (13.8 g/day). Cereal and cereal products were the main inulin contributors to percentage of total intake of group (58.1%). Next were mixed composite dishes (7.4%), vegetable, potatoes, beans group (7.4%), and fruits (8.5%). In conclusion, it is feasible to include inulin in the estimation of total dietary fibre. If inulin intake is assessed, total fibre consumption in children could increase by 8%, suggesting it is important to include inulin and fructans in fibre estimates of representative populations.

## 1 Introduction

Inulin is a non‐digestible soluble carbohydrate of β‐(2‐1) linked linear fructan chains with a wide degree of polymerisation (DP 2‐60). This makes it a versatile polysaccharide with a wide range of applications in the food industry but also an important dietary contributor to colonic substrates for the gut microbiota with several demonstrated health benefits (Roberfroid 2005). Although inulin is included in the definition of dietary fibre (DF) (Chambers et al. 2015), dietary surveys do not routinely include inulin and fructans in their fibre calculations (WHO/FAO 2009). This may be because the inclusion of inulin in the DF definition in the UK is relatively recent (Scientific Advisory Committee on Nutrition 2015). Also, it is difficult to measure inulin at the same time as other fibre components (McCleary 2023). This is due to differences in the physicochemical structure of the fibres found in the food matrix, which require specific analytical methods for their quantification. For example, the AOAC 985.29 method uses aqueous ethanol to precipitate insoluble, high molecular weight fibres such as cellulose, while inulin cannot be measured with the same method because of its low molecular weight and water‐soluble characteristics (McCleary 2023).

Inulin‐rich foods include chicory root and Jerusalem artichoke; it is also found in commonly consumed foods such as onion, garlic, bananas, leeks, and in much smaller quantities in widely consumed cereals like wheat, barley, and rye (Van Loo et al. 1995). Inulin extracts (from chicory root) have been used in food reformulation for sugar and fat replacement or added as prebiotics to functional foods (Qin et al. 2023). However, the extent of this contribution in the UK diet is not well established.

In the UK, breakfast represents an important contributor to children's energy and nutrient intake. Inulin‐containing foods like breakfast cereals and white bread contribute 20% to total energy and provide a quarter of total fibre intake in children (Gaal et al. 2018); in addition, industrially processed foods contribute 60%–65% of total energy intake in children (Rauber et al. 2019). Industrially processed foods, including breakfast cereals and low‐sugar/low‐fat reformulated foods such as confectionary, could be contributing to inulin consumption and thus to total DF intake (Biesiekierski et al. 2011). Other inulin‐containing foods such as onions and garlic are ingredients in commonly consumed ‘composite’ dishes such as pizza, pasta, and curries, and these contribute 8%–11% of energy intake in UK children aged 4–18 years. However, the contribution of inulin to total fibre intake from these foods is not measured. Thus, considering that only 14% of UK children aged 4–10 years and 4% of those aged 11–18 years meet DF recommendations (Public Health England 2020), the exploration of other possible sources of fibre in their diet is important.

In the UK, nationally representative estimates of fibre intake are based on the National Diet and Nutrition Survey (NDNS) data but the extent to which inulin has been included in the analysis is not clear. ‘McCance and Widdowson's The Composition of Foods’ is the food composition database used to calculate AOAC total fibre intake for the NDNS, but the specific contribution of inulin is uncertain (Public Health England 2021). When oligosaccharides are included, there is no differentiation between inulin and total starch, sugar, or fibre fractions. This is mainly because of limitations in the analytical methods used and the lack of differentiation for inulin‐only fractions. In addition, the inclusion of fructan‐type carbohydrate as part of DF analytes for food tables is not mandatory in many countries, including the UK, and depends on national authorities (de Menezes et al. 2013).

Inulin intake has not been well studied. Adult studies from the US (Continuing Survey of Food Intakes by Individuals [Moshfegh et al. 1999]) and Europe (Eurostat [Van Loo et al. 1995]) have used a small database to estimate inulin‐containing foods. A mean inulin intake of 4.0 g/day in UK adults has been described (*n* = 15) (Dunn et al. 2011) with 5 g/day in Belgium (*n* = 15) (Neyrinck et al. 2020). The main food sources of inulin in the Belgian diet were 43% from garlic/onions, 28% from vegetables, 20% from cereal products, and 8.7% from fruits. However, in the UK, their main food sources were wheat (66.2% ± 16.5%), then onion (17.3% ± 10.6%) and garlic (5.4% ± 7.3%). Studies reporting inulin intake in children are more scarce. A large US study (*n* = ~4400) estimated that children < 5 years consumed 1.34 g/day of inulin (range 0.55–2.13) and older children (6–11 years) consumed 2.21 g/day (range 0.90–3.52); mainly from wheat (69%), onion (23%), banana (3%), and garlic (3%) (Moshfegh et al. 1999). Thus, the inclusion of naturally occurring inulin in foods and isolated added inulin could help increase fibre intake. The aim of this study therefore was to test whether it is feasible to estimate the sources of inulin and how they contribute to total DF intake in a convenience sample of UK school‐age children.

## 2 Methods

### 2.1 Study Design and Sample Size

This was a feasibility, pilot cross‐sectional study using a convenience sample of 154 participants. The final sample size for this study was 148 children who completed the 24‐hour recall. When compared to the number of Glasgow primary school children in 2023 (*n* = 40 337) this sample provides an 8% margin of error at a 95% confidence level, which is considered acceptable for an exploratory study (Daniel 2012; Barlett et al. 2001).

### 2.2 Participants/Ethics

Children were recruited if the parents self‐reported that they were healthy and 5–12 years of age. Parents and children had to reside in Scotland and be English speakers. This research project (No. 200180150) was approved by the ethics committee of the MVLS College, University of Glasgow.

### 2.3 Recruitment

Children and their parents were recruited during science festivals and events held in community venues in Glasgow, UK, where parents and their children attended for leisure and educational purposes in 2019. Strategies to attract the interest of participants to take part in the research included the design of fibre‐related science activities that took into consideration the age of potential participants. At the end of each activity, parents were briefed about the study and if attendees were interested in taking part, the study was explained in detail, and they received a participant information sheet. Parents signed a consent form and children assented to take part in the study.

The event was also advertised by posters in wider community settings and on university notice boards, by word of mouth and via social media platforms including Facebook and Twitter/X. This research also used data from a pilot study where the aim was to explore the feasibility of using a breath hydrogen monitor in healthy children. As we recruited in venues with large child/parent attendance, this meant that this convenience sample was not representative of the broad population.

### 2.4 Dietary Fibre Assessment

Dietary fibre intake was estimated using one 24‐hour food recall with multiple pass questions conducted by one of three trained nutritionists who interviewed each child with parental help. Photographs of high fibre foods, including snacks and confectionery products, were used to help participants estimate food portions.

#### 2.4.1 Total Dietary Fibre Intake

Total fibre intake was calculated using Nutritics Research Edition v5.90 (Nutritics 2019) which contains data from the 7th Ed. of McCance & Widdowson food composition table (Public Health England 2021). This database uses the AOAC 985.29 which does not measure non‐digestible carbohydrates such as inulin (McCleary 2023).

The NDNS data Years 9–11 uses the AOAC method 985.29 and was used to compare our results to the representative sample of this age group.

#### 2.4.2 Inulin Intake

For the estimation of inulin intake, a list of foods containing inulin was collated from different sources and added to the Nutritics food database. First, a thorough search of published literature reporting fructan content of foods was conducted, and a list of foods containing inulin (g/100 g of product) was created using median/mean values reported (Biesiekierski et al. 2011; Neyrinck et al. 2020; Whelan et al. 2011). Available data on commercial products was also used. This was followed by the construction of a list of foods reported in the 24‐hour recall. The final list of inulin‐containing foods included breakfast cereals, breads, biscuits, potatoes, canned foods, and mixed composite dishes likely to contain inulin‐rich ingredients such as onion, garlic, and wheat. When specific food brands were mentioned, the manufacturer's website was used to obtain an updated ingredient list. When participants reported generic terms such as bread or cereal, standard white sliced bread and cornflakes were used to calculate intake. If no specific food brand was reported, information from the most popular brand was used (e.g., Heinz beans). Inulin content for UK breads was taken from (Whelan et al. 2011). For ‘homemade’ and ‘takeaway’ foods, the mean of three recipes from UK websites such as BBC Foods was used. A rule of three was used to estimate the inulin content of natural and commercial products.

### 2.5 Inulin Food Sources

Food sources of inulin were categorised in four groups. The cereal and cereal products group covered breads and breakfast cereals. The vegetables, potato, and beans group consisted of raw and cooked foods, including potatoes, carrots, and relevant canned soups. The fruit group included fruits and fruit juices/smoothies. The mixed composite dishes group included vegetable, meat, or fish‐based dishes.

### 2.6 Statistical Analyses

Descriptive statistics (mean, standard deviation, median, minimum, maximum, IQR, quartiles) were used for participants characteristics, inulin intakes, and food sources.

## 3 Results

Characteristics of children and their accompanying parents are described in Table 1. A total of 154 children were recruited for the activities. From these, 148 school‐aged children provided 24‐hour dietary recall and were included in the assessment of inulin intake/food sources. Median child age was 7 years (IQR: 5–12). The majority of children were from the least deprived areas (65%) of Scotland. Accompanying parents were mostly British (84%), female (73%) with a median age of 40 years (IQR: 26–69).

**TABLE 1:** Sociodemographic characteristics of children and accompanying parents.

Characteristic	*n* (%)
Children	
Sex	
Male	48 (38.7)
Female	76 (61.3)
Parent	
Sex	
Male	37 (26.8)
Female	101 (73.2)
Education level	
Up to high school/technician degree	41 (28.7)
Graduate/postgraduate degree	102 (71.3)
SIMD	
High deprivation (Q1, Q2)	42 (34.7)
Low deprivation (Q3, Q4, Q5)	79 (65.3)

Abbreviations: Q1–Q5, quartile 1 to quartile 5 of SIMD classification were Q1 is the lowest and Q5 the highest; SIMD, Scottish Index of Multiple Deprivation.

### 3.1 Inulin and Dietary Fibre Intake

**FIGURE 1:**
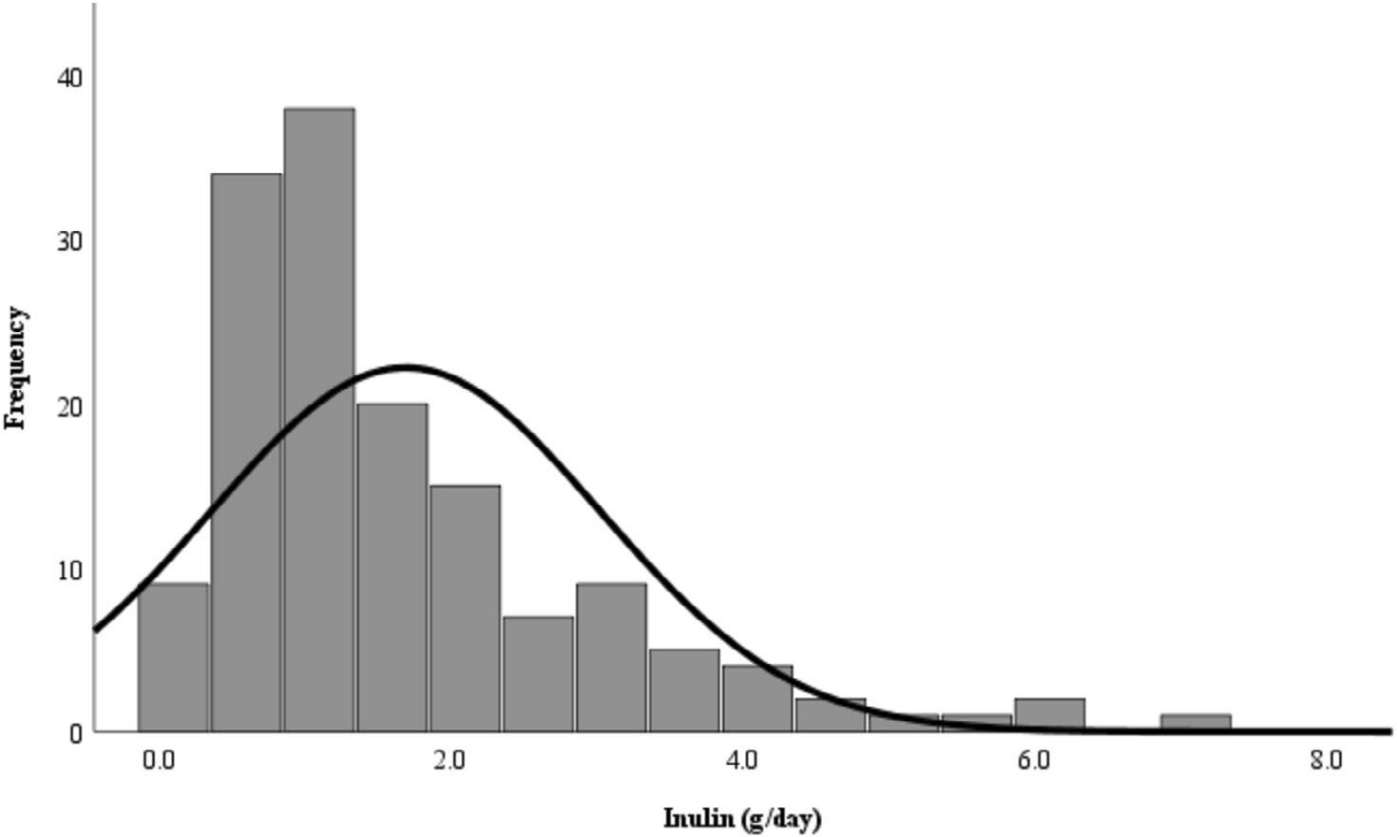
Inulin intake in school‐aged children. In healthy children (*n* = 148) assessed with one 24‐hour multiple‐pass recall, median inulin intake was 1.3 g/day (IQR: 0.1–7.1).

The distribution of inulin intake in this convenience sample was very variable and skewed to the left (Figure 1). Median inulin intake was 1.3 g/day (IQR: 0.1–7.1). The median total fibre was 14 g/day (IQR: 0.8–45.4). The median fibre‐plus‐inulin intake was 16.3 g/day (IQR: 11.8–21.1), which equates to an 8.5% contribution from inulin to total fibre.

### 3.2 Very High Inulin Consumers

Some high inulin consumers (P90, P1, P146, P2) consumed between 5 and 7 g/day. The sources of inulin were varied but did not include foods enriched with inulin.

- P90 (7 g/day): Shreddies, wholemeal bread, banana, spaghetti Bolognese, bagel, potato crisps.
- P1 (6 g/day): Cheerios multigrain, wholemeal bread, carrots, vegetable pakora, chicken korma.
- P146 (6 g/day): Weetabix, vegetable soup‐canned, cream crackers, spaghetti and meatballs in tomato sauce (containing onion, garlic).
- P2 (5 g/day): Oat flakes rolled, wholemeal bread, carrots, chicken korma, vegetable pakora.

### 3.3 Fibre Intake in Convenience Sample Versus NDNS

When the estimated amount of inulin (1.3 g/day) was added to the total DF content (14 g/day), the total intake (16.3 g/day) was comparable to the reported total fibre intake (15.2 g/day) of the older child group (11–18 years) in the NDNS survey (Table 2).

**TABLE 2:** Comparison of estimates in grams/day of inulin, fibre, and total fibre plus inulin intake via one 24‐hour dietary recall on school‐aged children versus total fibre intakes from NDNS.

	Inulin	Total fibre^a^	Total fibre^a^ plus inulin	Total fibre^a^‐NDNS (years 9–11)	
Age group	5–12 years old			4–10 years old	11–18 years old
Median	1.3	14	16.3	13.8	15.2
Q1	0.8	10.4	11.8	6.9	7.3
Q3	2.1	18.8	21.1	24.2	29.4
Minimum	0.1	0.8	0.9		
Maximum	7.1	45.4	46.5		

Abbreviation: NDNS, National Diet and Nutrition Survey.

^a^ Fibre estimated using AOAC 985.28 method.

### 3.4 Inulin Food Sources

The main foods contributing to inulin intake were the cereals and cereal products group (58.1%), followed by mixed composite dishes (19.1%), mainly due to onion and garlic content. This group consisted of takeaway/homemade meat and vegetable dishes (e.g., spaghetti Bolognese, chicken katsu curry). The third and fourth most contributing groups were the fruit group (8.5%), the vegetable, potatoes and beans group (7.4%), and other foods contributed 6.9% to inulin intake.

Detailed descriptions of the foods contributing to the inulin food sources by main food group are given in Tables S3–S6. The foods with the highest inulin contribution in each food group were white bread average, Weetabix, biscuits, spaghetti, bananas, carrots, baked beans, potato crisps, vegetable soup (canned), cheeseburger, and spaghetti Bolognese (due to garlic/onion content). No inulin‐supplemented foods featured in the food list.

## 4 Discussion

The aim of this study was to test the feasibility of estimating inulin intake, food sources, and its contribution to total DF consumption. We found that it was feasible to calculate inulin intake, its food sources, and its contribution to total fibre intake in a convenience sample of healthy school‐aged children. Inulin was found to contribute 8% to the total DF consumption levels. This is important because including inulin in the calculation of total fibre intake could reduce the apparent gap between DF recommendations and consumption levels. In addition, our findings demonstrate a need to measure inulin in foods and update food tables used to report fibre intake at the population level.

For example, if inulin is calculated in the UK NDNS survey, this would suggest an increment from 13.8 to 14.9 g/day in the age group 4–10 years (Public Health England 2020). Similarly, in the US, inclusion of inulin to total DF would suggest an increase from 14.7 to 16 g/day in children aged 6–11 years (U.S. Department of Agriculture ARS 2022). Although this is an important contribution to total fibre intake, this age group would be closer to the DF recommendations (20 g/day in the UK and 20–25 g/day in the US, respectively), but not by much.

For European children, DF recommendations are 14 g/day for 4–6 years and 16 g/day for those between 7 and 10 years. The inclusion of inulin at 8% would suggest higher consumption levels. For instance, this could lead to a 10% increase of total DF in Belgium and Portugal (European Commission 2021); 11% in Spain (Samaniego‐Vaesken et al. 2020); 8% in the Netherlands and 7% in Austria. However, the estimated levels of inulin contributing to total fibre intake will vary depending on the inulin‐containing foods consumed across European countries; for example, Mediterranean countries are more likely to consume onions and garlic due to their traditional diet patterns.

More locally, in Scotland, the mean DF intake in adolescent/young adults aged 16–24 years is 15.8 g/day and with the contribution of inulin it would increase by 8%, (~17 g/day) which is still well below recommendations, considering the DF recommendations for this age group (30 g/day) (Scottish Health Survey 2022).

Inclusion of inulin in foods, including supplementation, may provide specific benefits such as increased mineral bioavailability. Griffin and colleagues reported a 38% increase in colonic calcium absorption in a RCT of healthy girls supplemented with 8 g/day of inulin/oligofructose over 3 weeks (Griffin et al. 2002). In another crossover trial, with girls and boys, an 8.5% increase in colonic calcium absorption was reported after 1 year supplementation with 8 g/day of inulin‐type fructan (Abrams et al. 2005). This increase in colonic mineral absorption with inulin is mainly mediated by the fermentability of DF. For example, DF with a short‐chain length is more fermentable than long‐chain (e.g., oligofructose vs. inulin). The mechanism of action is influenced by the production of short‐chain fatty acids (SCFA) which lowers colonic pH. This leads to the solubilisation of calcium facilitating its colonic absorption (Roberfroid 2007).

Inulin can also impact gut microbiota composition due to its prebiotic effect. A prebiotic is ‘a substrate that is selectively utilised by host microorganism conferring a healthy benefit’ (Gibson et al. 2017). Thus, inulin promotes the growth of gut microbiota considered beneficial (including *Lactobacillus* and *Bifidobacterium*) (Lozupone et al. 2012).

Inulin may also increase daily stool frequency. In a crossover, RCT children aged 4–6 years with simple constipation consumed snacks and beverages enriched with 1.8–3.4 g of pea hull fibre and 5 g of inulin over 3 weeks. Daily stool frequency increased at week 2 (0.54 ± 0.18) and week 3 (0.67 ± 0.22) (Flogan and Dahl 2010).

Inulin supplementation may also have a role in energy intake and appetite regulation in healthy children and those with overweight and obesity (OW/OB). For example, in young children (4–6 years), a reduction in energy intake (~134 ± 11 kcal/day) was reported after consumption of a snack/beverage enriched with pea hull fibre and 5 g of inulin (Flogan and Dahl 2010). Similarly, a parallel RCT with older, OW/OB children (7–12 years), reported a reduction in energy intake of ~113 ± 72.7 kcal/day in the 11–12 years old children after consumption of 8 g/day of oligofructose enriched inulin over 16 weeks compared to 137 ± 77.4 kcal/day in the placebo group, *p* = 0.04 (Hume et al. 2015). The same study reported a significant reduction in prospective food consumption and increased fullness ratings and fasting appetite hormones ghrelin and adiponectin in the children consuming inulin after 16 weeks (Hume et al. 2015). The surprisingly higher levels of ghrelin were attributed to appetite adaptations in subjects with obesity.

The mechanisms for appetite regulation resulting from inulin consumption are likely mediated by SCFA production which stimulates the release of appetite hormones (ghrelin, GLP‐1, PYY) via free fatty acid receptor‐2 (FFAR2) often by the ‘second meal effect’ (Chambers et al. 2015; Ibrügger et al. 2014). This may also delay gastric emptying. In a crossover trial in older children aged 18 years after consumption of pasta enriched with 11 g/day of inulin over 5 weeks, there was a positive correlation between the gut hormone plasma somatostatin and half‐emptying time (Russo et al. 2011).

Inulin can be found in high concentrations in foods such as Jerusalem artichoke, chicory root, salsify, and garlic (between 15.6 and 16.7 g/100 g) (Van Loo et al. 1995). However, none of these foods were consumed by the population in this study. In contrast, foods contributing to inulin intake were onions and garlic in mixed composite meals. A contributor to inulin intake was white bread, which contains 0.86 g/100 g inulin/food, while a much lower contribution was observed from the vegetables, potatoes, and beans group and the fruit group.

The inulin intake levels (1.3 g/day) in our study were within the range of inulin consumed by school‐aged US children (0.9–3.5 g/day) in a larger (*n* = 1432) study using secondary data from a national representative survey of children (< 5–11 years) (Continuing Survey of Food Intakes by Individuals) (Moshfegh et al. 1999) and similar inulin food sources as in our cohort were reported. Wheat (69%) was the main ingredient in the grain and cereal products, followed by onions (23%) and garlic (3%), which were also common in our study as ingredients within the mixed composite dishes group.

A main limitation of our data is the feasibility and pilot nature of the study design as well as the use of only one 24‐hour dietary recall. This method is prone to recall bias, uncharacteristic of the usual diet, and participants may report socially desirable foods (Foster and Bradley 2018; Livingstone and Robson 2000). This could explain the maximum inulin intake level (7.1 g/day) and the maximum total dietary fibre intake (46 g/day). However, we attempted to minimise this by using food photos of fibre‐rich foods and parental help (for younger children). Still, these data are not from a random day. This one 24‐hour dietary recall collected data from meals eaten on Friday, Saturday, and Sunday. This allowed us to have a range of meals that children ate on these days. Also, recipes that contained inulin‐rich foods such as onions/garlic were described by parents when asked.

The food sources and intake levels reported in our study are not representative of the Scottish population because of the high socioeconomic level of our sample. This might differ from the consumption levels of other segments of the population, as suggested in the US survey, where participants from the highest income category consumed more (2.8 g/day) inulin versus those in middle/lowest income levels (2.3–2.5g/day) (Moshfegh et al. 1999). These differences in inulin consumption by deprivation level are important. In the Scottish Health Survey 2019, it was reported that high fibre/low sugar cereals (a source of inulin) consumed at least 5 days a week are much higher (37%–39%) in the least deprived group (SIMD Q4–Q5) versus the most deprived sectors (SIMD Q1–Q2) with 18%–22% (Scottish Health Survey 2020).

Another limitation was the time of data collection, as this study was conducted only between Summer and Autumn. This may have influenced the types/amounts of inulin‐rich foods consumed. For example, a higher consumption of vegetables and cereals was reported in Spring/Summer (Stelmach‐Mardas et al. 2016) compared to a higher consumption of fruits and cereals in the Autumn/Winter season. Finally, it is a limitation of our approach to estimate total fibre intake from data obtained using dietary analysis software plus added inulin as estimated from our study when it is not completely clear whether the methods used to estimate fibre intake in the diet software included inulin measurements in the fibre content or not.

### 4.1 Implications

For national governments and researchers, the main implication is the update of the fibre intake. They should consider the potential contribution of inulin to total fibre reported in the NDNS. This is important because it can contribute to a more accurate estimation of average fibre intakes, which are also used as a basis for the current DF recommendation. Also, this study may encourage updating the food composition tables used in national surveys and conducting future food analysis with the latest methodology of AOAC fibre, which can allow better calculation of fibre types and hence total fibre intake (McCleary 2023).

In addition, inulin is mostly consumed from sources with low inulin concentrations (e.g., white bread average). This may suggest an opportunity to increase the amount of non‐digestible oligosaccharides in such foods and promote other sources such as vegetables/fruits, especially onions and leeks. These are present in many dishes eaten by young children, including soups, stews, pizzas, pasta, and curry sauces. Onions and leeks add flavour to many dishes, and this could also help maintain preference for vegetable‐rich foods in later life.

## 5 Conclusions

It is feasible to estimate inulin intake levels and food sources in school‐aged children. Incorporation of inulin in total fibre values could make an important contribution of 1.3 g/day to the current DF consumption levels and move closer to national recommendations. To fully estimate the contribution of inulin and other non‐digestible oligosaccharides, it is important to include these in national food composition tables and dietary analysis tools and software to be used in future studies using representative population samples.

## Author Contributions

Gabriela Morillo‐Santander planned the study, conducted the acquisition of data, undertook the analyses, data interpretation, and produced the first draft. Christine Ann Edwards advised and supervised study design, data analysis, and data interpretation and provided extensive feedback on the manuscript. Ada Lizbeth Garcia advised on study design, data analysis, and data interpretation, supervised the initial write‐up, and provided feedback on the manuscript. All authors approved the final manuscript as submitted and agree to be accountable for all aspects of the work.

## Conflicts of Interest

Christine Ann Edwards has chaired ILSI Europe groups on colonisation of the infant gut. Gabriela Morillo‐Santander has no conflicts of interest to declare. Ada Lizbeth Garcia has no conflicts of interest to declare.

## Supporting information


**Data S1.** nbu70022‐sup‐0001‐Tables.docx.

## Data Availability

The data that support the findings of this study are available from the corresponding author upon reasonable request.
