# Supplementary material for: Estimating Inulin Intake and Its Contribution to Total Fibre Intake in UK School‐Aged Children: A Pilot Feasibility Study
Source: Nutr Bull. 2025 Aug 13;50(4):633–9. doi: 10.1111/nbu.70022 (PMC12621174; doi:10.1111/nbu.70022)
Supplement: Supplementary file 1 — Data S1. nbu70022‐sup‐0001‐Tables.docx. [file NBU-50-633-s001.docx]

**Supplementary tables**

**Table 3. Food sources of inulin on the cereal and cereal products group based on one 24-h recall in school-aged children.**

| **Food** | **% contribution to total inulin intake** | **Mean** | **SD** |
| --- | --- | --- | --- |
| **Breads total** | **36.4** |  |  |
| Rye Bread | 0.8 | 0.9 | - |
| Bagel, wholemeal | 0.3 | 0.7 | - |
| Bagels, plain | 1.2 | 0.7 | 0.2 |
| Bread rolls, malted wheat | 2.4 | 0.7 | 0.1 |
| Bread rolls, white, crusty | 6.2 | 0.7 | 0.3 |
| Bread rolls, white, soft | 1.8 | 0.9 | - |
| Bread, brown, average | 2.8 | 0.6 | 0.2 |
| Bread, pitta, white | 0.3 | 0.4 | - |
| Bread, seeded | 0.1 | 0.3 | - |
| Bread, white, average | 10.5 | 0.6 | 0.1 |
| Bread, white, French stick | 0.4 | 0.6 | - |
| Bread, white, sliced | 2.6 | 0.5 | 0.1 |
| Bread, white, toasted | 1.4 | 0.4 | 0.2 |
| Bread, white, with added fibre | 1.3 | 0.3 | 0.1 |
| Bread, wholemeal, average | 4.2 | 0.6 | 0.2 |
| **Breakfast cereals total** | **13.3** |  |  |
| Breakfast cereal, Cornflakes | 1.2 | 0.2 | 0.1 |
| Breakfast cereal, Cornflakes, frosted | 0.1 | 0.3 | - |
| Breakfast cereal, Crunchy/crispy muesli | 0.2 | 0.6 | - |
| Breakfast cereal, Shreddies | 2.6 | 0.8 | 0.2 |
| Breakfast cereal, Cheerios | 0.3 | 0.2 | 0.1 |
| Breakfast cereal, Coco Pops | 1.0 | 0.4 | 0.2 |
| Breakfast cereal, Rice Krispies | 1.4 | 0.3 | 0.2 |
| Breakfast cereal, Shredded wheat type | 0.7 | 0.8 | 0.2 |
| Breakfast cereal, Weetos, Nesquick | 0.3 | 0.4 | - |
| Breakfast cereal, Weetabix Crispy Minis | 0.2 | 0.5 | - |
| Breakfast cereal, Weetabix | 4.4 | 0.7 | 0.3 |
| Muesli | 0.5 | 0.4 | - |
| Oat flakes, rolled | 0.4 | 0.1 | - |
| **Biscuits total** | **5.2** |  |  |
| Biscuits, cookies, chocolate chip | 0.6 | 0.6 | 0.4 |
| Biscuits, digestive, half coated in chocolate | 0.4 | 0.3 | - |
| Biscuits, digestive, plain | 1.0 | 0.3 | 0.1 |
| Biscuits, half coated in chocolate | 1.1 | 0.4 | - |
| Cereal bars, with fruit and/or nuts | 0.8 | 0.6 | 0.3 |
| Cream crackers | 0.4 | 0.3 | 0.1 |
| Oatcakes, Sainsbury, Nairns | 0.0 | 0.1 | - |
| Shortbread | 0.2 | 0.3 | - |
| S’mores, Tesco | 0.1 | 0.3 | - |
| **Pasta total** | **3.2** |  |  |
| Pasta, spaghetti, canned, in tomato sauce | 0.1 | 0.3 | - |
| Pasta, white, dried, boiled in unsalted water | 3.1 | 0.4 | 0.2 |

Standard deviation in some foods was 0.0 and this was not account for in the tables.

**Table ‎**4**. Food sources of inulin in the vegetables, potatoes and beans group, based on one 24-h recall in school-aged children.**

| **Food** | **%** | **Mean** | **SD** |
| --- | --- | --- | --- |
| **Vegetables total** | **1.6** |  |  |
| Beetroot, boiled in unsalted water | 0.1 | 0.2 | - |
| Carrots, old, boiled in unsalted water | 1.0 | 0.2 | 0.1 |
| Carrots, old, raw | 0.2 | 0.1 | 0.1 |
| Carrots, young, raw | 0.2 | 0.5 | - |
| Peppers, capsicum, red, raw | 0.1 | 0.1 | 0.1 |
| **Legumes total** | **1.3** |  |  |
| Baked beans, canned in tomato sauce | 1.0 | 0.20 | 0.13 |
| Beans, green, boiled in unsalted water | 0.1 | 0.12 | 0.06 |
| Beans, red kidney, canned in water | 0.1 | 0.16 | - |
| Chickpeas | 0.1 | 0.26 | - |
| **Canned soups total** | **2.5** |  |  |
| Soup, lentil, Heinz | 0.1 | 0.1 | - |
| Soup, vegetable, canned | 2.4 | 2.9 | 0.2 |
| **Potatoes total** | **2.0** |  |  |
| Potato crisps, fried in sunflower oil | 2.0 | 0.1 | - |

Standard deviation in some foods was 0.0 and this was not account for in the tables.

**Table 5. Food sources of inulin in the fruit group based on one 24-h recall in school-aged children.**

| **Food** | **%** | **Mean** | **SD** |
| --- | --- | --- | --- |
| **Fruits total** | **7.6** |  |  |
| Bananas, flesh only | 5.8 | 0.7 | - |
| Fruit salad, homemade | 0.1 | 0.2 | - |
| Melon, flesh only, average | 0.7 | 0.2 | 0.1 |
| Watermelon, flesh only | 0.2 | 0.5 | - |
| Nectarines, flesh, and skin | 0.1 | 0.2 | - |
| Peaches, raw, flesh and skin | 0.7 | 0.9 | 0.6 |
| **Fruit smoothies total** | **0.9** |  |  |
| Smoothie, Innocent, Strawberries & Bananas, | 0.3 | 0.3 | - |
| Smoothie, Innocent, Strawberries, Blackberries | 0.5 | 0.3 | 0.1 |
| Smoothie, Strawberries & Bananas | 0.1 | 0.3 | - |

Standard deviation in some foods was 0.0 and this was not account for in the tables.

**Table 6. Contribution of inulin to the mixed composite dishes group, based on one 24-h recall in school-aged children.**

| **Food** | **%** | **Mean** | **SD** |
| --- | --- | --- | --- |
| **Meat/Vegetable dishes total** | **19.1** |  |  |
| Chilli con carne, homemade | 1.1 | 2.6 | - |
| Meatballs, pork, and beef, in tomato sauce | 1.6 | 1.5 | - |
| Spaghetti Bolognese, homemade | 2.4 | 3.0 | 1.4 |
| Macaroni cheese, homemade | 0.3 | 0.8 | - |
| Black pudding, dry-fried | 0.2 | 0.5 | - |
| Chicken goujons, premium, grilled/baked | 1.1 | 1.4 | 0.4 |
| Chicken Kiev, frozen, baked | 0.3 | 0.7 | - |
| Fish fingers, cod, grilled/baked | 0.5 | 0.1 | - |
| Burger, Big Mac | 0.9 | 2.3 | - |
| Burger, cheeseburger | 2.7 | 1.4 | 0.4 |
| Burger, chicken | 0.3 | 0.8 | - |
| Burger, hamburger, takeaway | 1.4 | 1.2 | - |
| Broth Soup | 0.5 | 1.1 | - |
| Chicken Katsu Curry | 0.5 | 1.4 | - |
| Chicken Korma | 2.1 | 2.6 | - |
| Homemade Veggie Burger | 0.7 | 1.8 | - |
| Pakora, vegetable | 1.0 | 2.5 | - |
| Soup, vegetable, homemade | 0.6 | 1.4 | - |
| Vegetable dumplings | 1.0 | 2.5 | - |

Standard deviation in some foods was 0.0 and this was not account for in the tables.
